# Wheat V-H^+^-ATPase Subunit Genes Significantly Affect Salt Tolerance in *Arabidopsis thaliana*

**DOI:** 10.1371/journal.pone.0086982

**Published:** 2014-01-30

**Authors:** Xiaoliang He, Xi Huang, Yinzhu Shen, Zhanjing Huang

**Affiliations:** 1 College of Life Science of Hebei Normal University, Shijiazhuang, Hebei, China; 2 School of Biological Science and Engineering, Hebei University of Science and Technology, Shijiazhang, Hebei, China; University of Nottingham, United Kingdom

## Abstract

Genes for V-H^+^-ATPase subunits were identified and cloned from the salt-tolerant wheat mutant RH8706-49. Sequences of these genes are highly conserved in plants. Overexpression of these genes in *Arabidopsis thaliana* improved its salt tolerance, and increased the activities of V-H^+^-ATPase and Na^+^/H^+^ exchange, with the largest increase in plants carrying the c subunit of V-H^+^-ATPase. Results from quantitative RT-PCR analysis indicated that the mRNA level of each V-H^+^-ATPase subunit in the *Arabidopsis* increased under salt stress. Overall, our results suggest that each V-H^+^-ATPase subunit plays a key role in enhancing salt tolerance in plants.

## Introduction

Salt stress is a major factor adversely affecting the growth of plants. It can cause an elevation of Na^+^ concentration in the cytoplasm, which results in osmolarity stress and ion poisoning [1]. Salt stress can also cause large reductions in yield. Thus, increasing the salt tolerance of plants is of great interest in agriculture.

H^+^-ATPases are found in the plasma membrane and various endomembrane systems. They are essential for establishing the cross-vacuolar-membrane proton gradient, which promotes vacuole Na^+^ compartmentalization and plant salt tolerance. There are two functional categories of H^+^-ATPases. The first category of H^+^-ATPases, such as F-type H^+^-ATPase, synthesize ATP using cross-membrane chemical potential. The second category of H^+^-ATPases, such as P-type and V-type H^+^-ATPases, hydrolyze ATP to generate cross-membrane proton potential. In the *Suaeda salsa*, enhancement of V-H^+^-ATPase expression is considered to be the major mechanism for salt resistance. Enhanced V-H^+^-ATPase expression provides essential energy for cross-membrane ion transport in vacuole [2]. The increased activity of V-H^+^-ATPase was found not to result from structural changes in this enzyme, but from an increase in its gene expression [3].

High salt concentration can increase the mRNA level of vacuolar V-H^+^-ATPase [4]. In *Mesembryanthemum crystallinum*, salt treatment increased the transcription of A, B, E, F, c and G subunit mRNAs [5]–[7]. Salt-stressed and salt-adapted tobacco suspended cell culture also showed an increased level of A subunit mRNA [8]. In *Beta vulgaris* L. and its suspended cell culture, NaCl stimulated coordinated changes of A and c subunits [9]–[10]. In *S. salsa* (L.) Pall, salt stress increased the transcription and translation of B and c subunits [11]. *SaVHAc1*, *LbVHA-c1* and *ThVHAc1* enhance salt tolerance in transgenic plants [12]–[14]. We previously obtained the sequences of E and B subunits, and found that salt treatment increased the expression of these two subunits. Overexpression of these subunits in wild-type *Arabidopsis thaliana* increased salt tolerance of transgenic *Arabidopsis thaliana* [15]–[16].

Here, we have cloned the other subunits besides E and B from a wheat salt-tolerant mutant (RH8706-49) using RT-PCR and overexpressed these subunits in *Arabidopsis thaliana* to study their role in salt tolerance.

## Materials and Methods

### Plants

Wheat salt-tolerant mutant RH8706-49 and *Arabidopsis thaliana* Columbia were used in this study.

### Microarray Analysis

Plants of the salt-tolerant wheat line RH8706-49 were treated with 135 mM NaCl for 0, 1, 6, 12, 72 h, respectively. The roots were then taken for total RNA preparation using the TRIzol (Invitrogen) reagent. Total RNA was purified with the RNeasy Mini kit (Qiagen). Double-stranded cDNA was synthesized with the one-cycle cDNA Synthesis Kit (Affymetrix), and then purified with the GeneChip Sample Cleanup Module (Affymetrix). The purified cDNA was used to prepare biotin-labeled cRNA using a GeneChip IVT Labeling Kit, according to the manufacturer’s instructions. The biotin-labeled cRNA was fragmented at 94°C for 35 min, which yielded the targets used for hybridization. The targets were hybridized with the Affymetrix Wheat Genome Array P/N:520254, and washing and scanning were carried out according to the assay procedure. The hybridization image was analyzed with Affymetrix Microarray Suite 5.0 software and the data were normalized. Clustering analysis was carried out with the Cluster and Tree View software.

### Cloning of Wheat V-H^+^-ATPase Genes

Total RNA was extracted from the RH8706-49 plants at the second leaf stage. cDNA synthesis was carried out as described previously [17]. The full length cDNA sequence was obtained and primers were designed using the Primer Premier 5.0 software (Table S1).

### Binary Expression Vector Construction and Transfection of *Arabidopsis thaliana*

The full-length cDNAs encoding V-H^+^-ATPase subunits were amplified by PCR using specific primers (Table S2) and inserted into the binary vector, pCAMBIA1300, under the control of the CaMV 35S promoter. The expression vector was then introduced into *Agrobacterium tumefaciens* (GV3101) using the freeze-thaw method. The transformed *Agrobacterium* was used to transform Arabidopsis thaliana [18]–[20]. Transgenic Arabidopsis seeds were screened using Hygromycin (25 mg/L) that was added to the MS medium [21]. Further RT-PCR validation of the selected transgenic plants was performed (1).

### Salt Tolerance Test

After surface disinfection, seeds of the wide type and three homozygous transgenic lines were placed on MS medium that contained 0 or 70 mM NaCl and cultured in a 22°C light incubator (16 h light, 8 h dark). Root length was measured at 7 d. The plants cultured on MS media for 5 d were transferred to media containing vermiculite and cultured at 22°C (16 h light, 8 h dark), with Hoagland’s solution as a fertilizer. One week later, plants were treated with Hoagland’s solution every 4 d containing either 0 or 100 mM NaCl until 16 d. The plants were then examined.

### V-H^+^-ATPase and Na^+^/H^+^ Exchange Activity Determination

After culture for 5 d on MS, *Arabidopsis thaliana* plants were transplanted to potting soil containing vermiculite. After growth at 22°C for 30 days, the shoots were used for preparation of vacuolar membrane vesicles, and for V-H^+^-ATPase and Na^+^/H^+^ exchange activity tests. Vacuolar membrane vesicles were prepared as described previously [22] with slight modification. Ten grams of plant material was ground in liquid nitrogen, mixed (1∶3 W/V) with homogenizing buffer [25 mM Tris/MES, pH7.5, 250 mM sucrose, 0.5% (W/V) BSA, 10% (V/V) glycerol, 1 mM PMSF, 5 mM EGTA, 2 mM DTT, 0.6% (W/V) PVPP] and filtered with four layers of gauze. The filtrate was spun once at 480 g for 10 min. Then, the supernatant was obtained and spun once at 6,000 g for 10 min to remove the precipitate. The supernatant obtained by this centrifugation was again centrifuged at 60,000 g for 30 min, and the obtained precipitate was resuspended in suspension buffer (5 mM Tris/MES, pH 7.5, 10% glycerol, 250 mM sucrose). The suspension was then carefully overlaid on a series (8%, 25%, 40% W/V) of sucrose density gradient and spun at 70,000 g for 2 h. The component within the range of 8%–25% in the sucrose gradient was collected for further analysis. For specific measurement of the V-H^+^-ATPase activity, Na_3_VO_4_ and NaN_3_, inhibitors of plasma membrane H^+^-ATPase and mitochondrial H^+^-ATPase, respectively, were added to the reaction buffer [15]. The proton transport activity of H^+^-ATPase was determined through acridine orange Xuorescence quenching with a Hitachi F-2500 spectroXuorometer (excitation at 490 nm, emission at 525 nm) [23]. The measuring buffer contained 10 mM MES/Tris (pH 7.5), 250 mM sorbitol, 5 µM acridine orange, 50 mM KCl, 3 mM MgSO_4_ and 20 µg of membrane protein. The reaction was initiated by the addition of 3 mM ATP. The Na^+^/H^+^ exchange activity was inferred from the Na^+^-induced dissipation of a preformed pH gradient maintained by the activity of the V-H^+^-ATPase. The reaction media were the same as those used for H^+^ transport assays. After the fluorescence quenching had reached a steady state, aliquots of the desired salt solution were added, and the initial rates of fluorescence recovery were determined during the first 15 s. Initial rates of Na^+^-dependent fluorescence recovery represented the activity of Na^+^/H^+^ antiport (expressed as Δ% F min^−1^ mg^−1^ protein). The selectivity of the Na^+^/H^+^ antiport was evaluated by adding various salts to dissipate the pH gradient in vacuolar membrane vesicles.

### Expression of V-H^+^-ATPase in *Arabidopsis thaliana*

*Arabidopsis thaliana* plants grown for 30 d were treated with 170 mM NaCl for 0, 1, 6, 12, 24 and 72 h. Then, total RNA was extracted and cDNA was synthesized using the methods described above. Real-time quantitative PCR was conducted using Rotor Gene-3000 Advanced PCR equipment. The software driving the equipment is RG3000 6.0 (Corbett Research, Australia). β-actin (GenBank accession No. AB181991) was used as an internal control. PCR product was detected using SYBR Green I (Macroprobe). The primers are listed in Table S3. Amplification and melting curve analysis were performed. Quantitative measurement was carried out by the comparative Ct method [24].

## Results

### Cloning of Wheat V-H^+^-ATPase Gene

**Figure 1:**
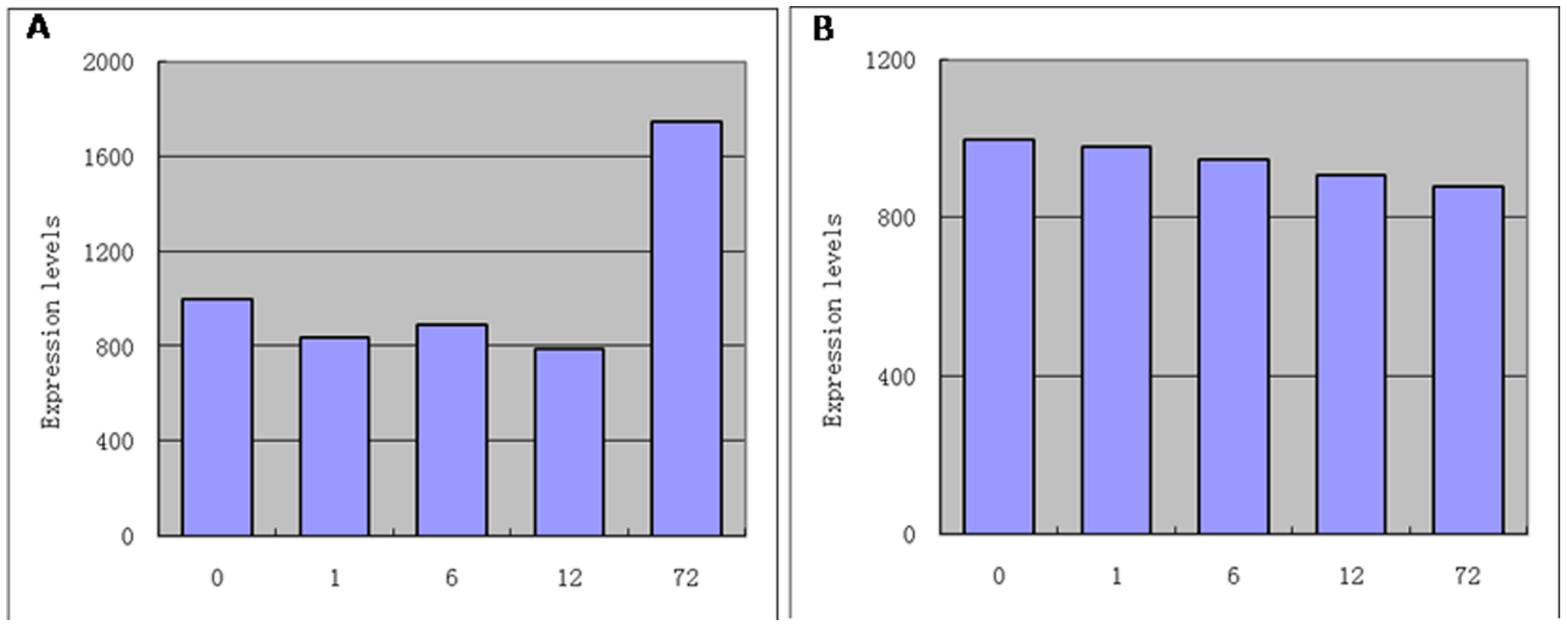
The expression of the probe after exposure to salt stress for 0-tolerant wheat mutant RH8706-49. A. CD880816; B. AK332225.

Gene chip technology was used to study the overall gene expression of RH8706-49 under salt stress and to obtain differential gene expression patterns for 61,215 wheat probes. The expression for one probe (gb: CD880816) is shown in Fig. 1A. After 12 h of salt stress, its expression was up to 2.2 times higher than that in the untreated control. We selected this probe and cloned the full-length cDNA sequence of the probe by RT-PCR. This gene contained 2466 bp of a complete open reading frame encoding an unknown protein (821 amino acids) and was named a subunit of V-H^+^-ATPases. The sequences were recorded in the GenBank database with accession numbers JN033547. We used a same method to obtain sequences D subunit of V-H^+^-ATPases by the probe AK332225 (Fig. 1B) and the sequences were recorded in the GenBank database with accession numbers JN107805. We had obtained sequences for subunits A (DQ432014), c (DQ631550), C (DQ631548), d (DQ631549), F (DQ486058), G (DQ491026) and H (DQ681104).

### Phylogenetic Analysis

**Figure 2:**
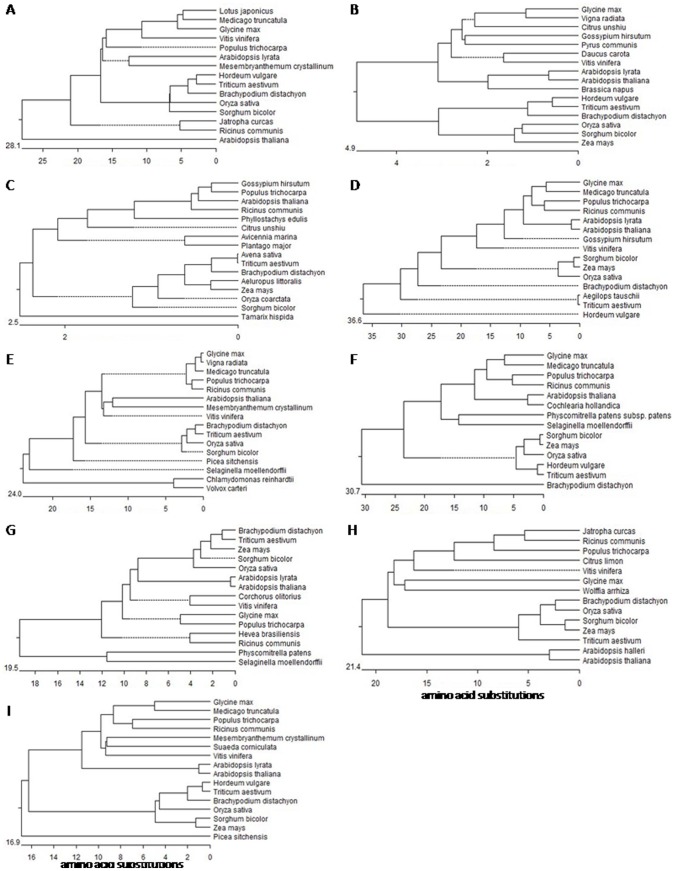
Phylogenetic trees for different V-H+-ATPase subunit genes, including wheat genes. A, subunit a; B, subunit A; C, subunit c; D, subunit C; E, subunit d; F, subunit D; G, subunit F; H, subunit G; and I, subunit H.

Phylogenetic analyses were carried out for each subunit. The results indicate that each of the subunits is very similar in sequence to its homologs in other species. The c subunit has the highest sequence conservation, followed in descending order by A, H, F, G, d, a and D. The least conserved is the C subunit (Fig. 2).

### Salt Tolerance of Transgenic Plants

**Figure 3:**
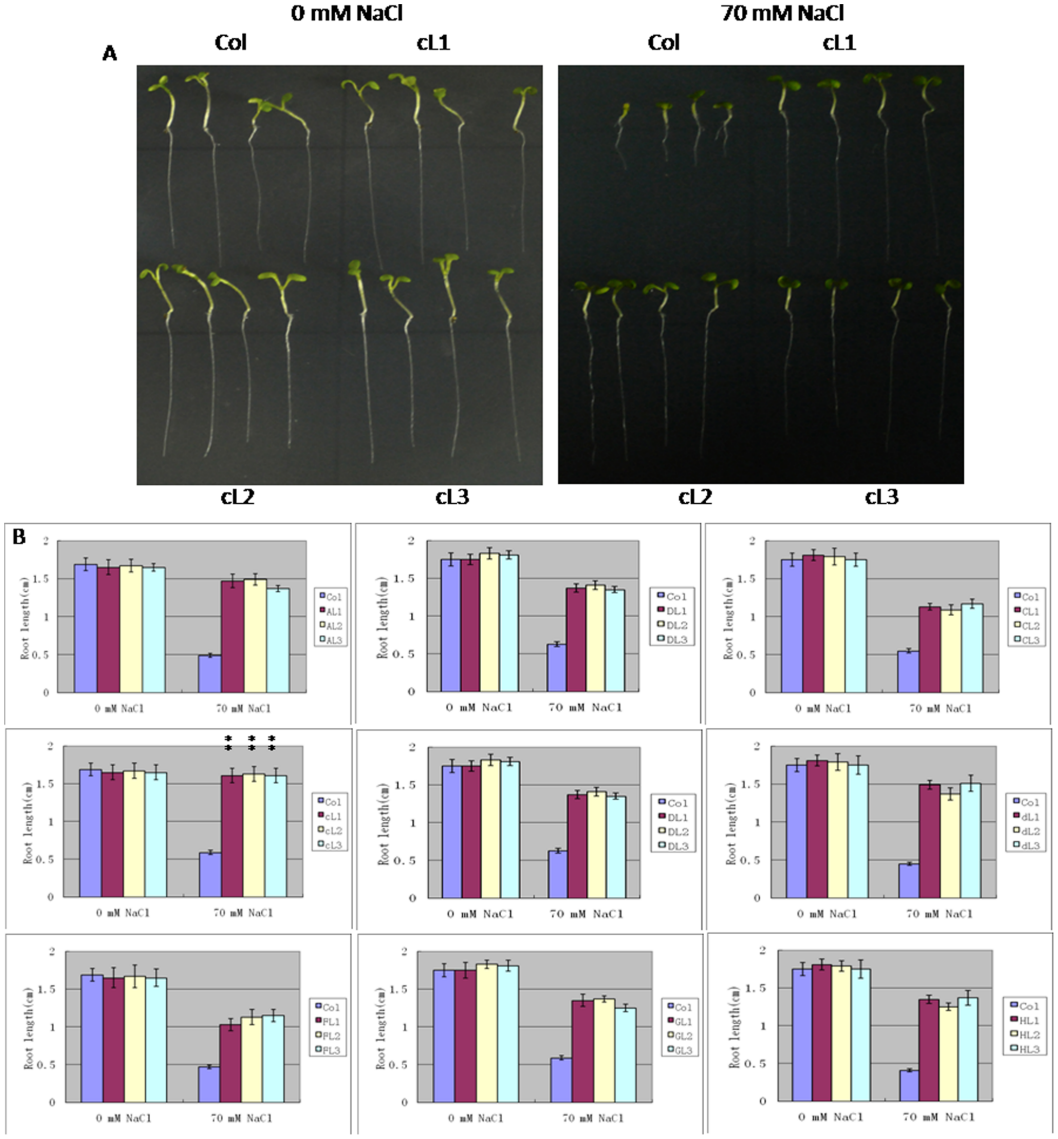
Phenotype of root growth in transgenic carrying A, a, C, c, D, d, F, G and H subunit and wild-type *Arabidopsis thaliana* plants. Seeds of transgenic (L1, L2, L3) and wild-type plants (Col) were germinated on MS agar medium containing 0 or 70 mM NaCl for 7 d. Values are the means ± SD (n = 5) (Student’s t-test, P<0.05).

**Figure 4:**
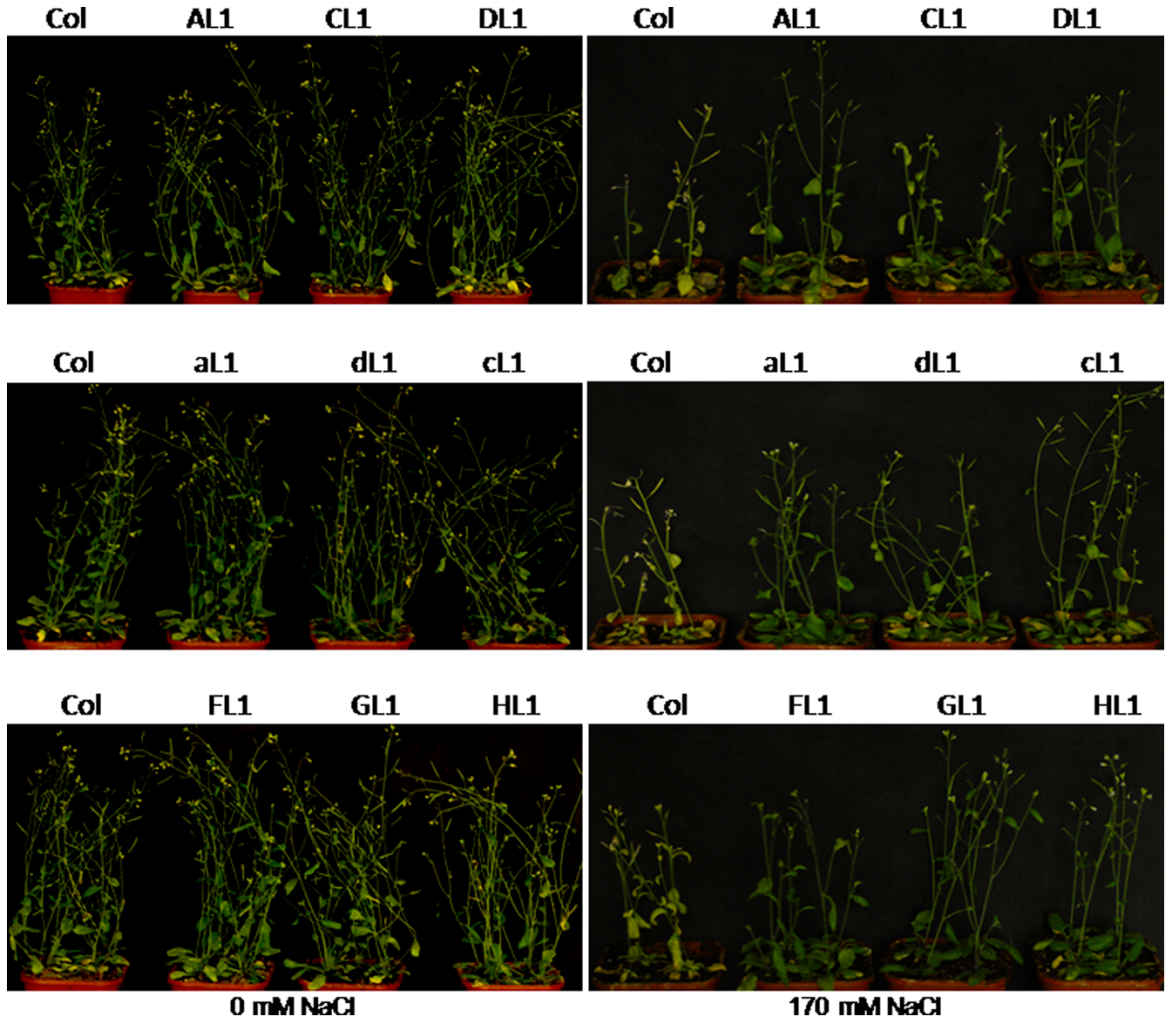
Phenotype of transgenic carrying A, a, C, c, D, d, F, G and H subunit and wild-type *Arabidopsis thaliana* plants. Seeds of transgenic (L1) and wild-type plants (Col) grown on MS medium for 5 d were transplanted into the growth media containing a mixture of vermiculite. One week later, the seedlings were watered with Hoagland’s solution containing 0 or 170 mM NaCl for 16 d.

The salt tolerance of homozygous plants (L1, L2 and L3) carrying a V-H^+^-ATPase subunit transgene was tested, using wild-type plants as a control. In MS media [20] with 0 mM NaCl, transgenic plants and their wild-type counterparts showed no difference in terms of growth. In media containing 70 mM NaCl, the transgenic plants had clearly longer roots than their wild-type counterparts. Roots of transgenic plants harbouring c, A or H subunit were 2.5-fold longer than that of the controls (Student’s t-test, P<0.01). Roots with the C subunit were twofold longer than those in controls (Fig. 3) (Student’s t-test, P<0.05). Transgenic plants showed a better overall shape than controls, and could bloom and bear seeds normally, while the control wilted and became scorched (Fig. 4). These results suggest that V-H^+^-ATPase could substantially increase the salt tolerance of the transgenic plants.

### V-H^+^-ATPase and Na^+^/H^+^ Exchange Activities

**Figure 5:**
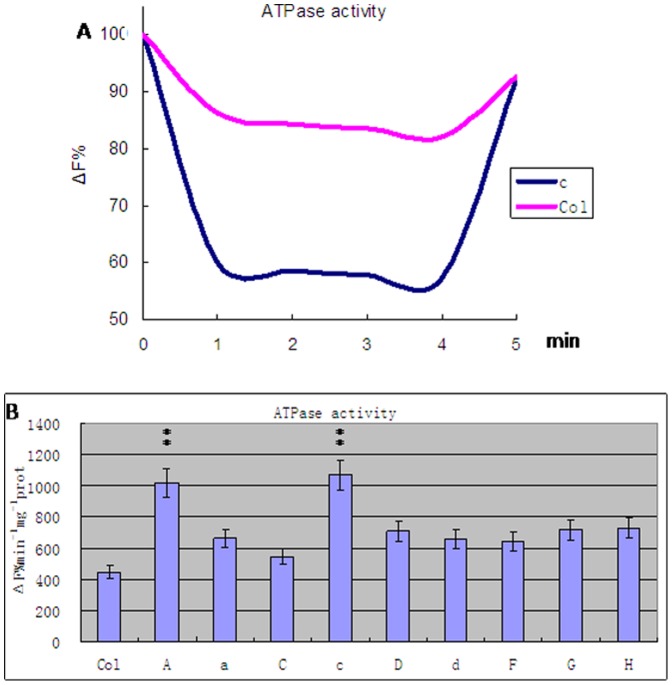
Activity of V-H^+^-ATPase in transgenic carrying A, a, C, c, D, d, F, G and H subunit from wheat and wild type *Arabidopsis thaliana* plants(Col). A. The accumulation of protons inside the vesicles is determined through acridine orange Xuorescence quenching. B. The activity of V-H^+^-ATPase. Values are means ± SD (n = 5 independent experiments) (ANOVA test, P<0.01).

Vacuolar membrane vesicles were prepared from transgenic and wild-type plants and tested for V-H^+^-ATPase activities (Fig. 5, Fig. S2). The results indicate that transgenic plants had significantly higher V-H^+^-ATPase activities (ANOVA test, P<0.01). Plants carrying the c or A subunit exhibited activities twofold those of controls and transgenic plants with other subunits (Least significant difference test, P<0.01). Plants with the C subunit transgene exhibited the smallest increase in activity: 1.2-fold that of the controls (Least significant difference test, P<0.05). The remainder of the transgenic plants had activities 1.5-fold higher than the controls (Least significant difference test, P<0.05).

**Figure 6:**
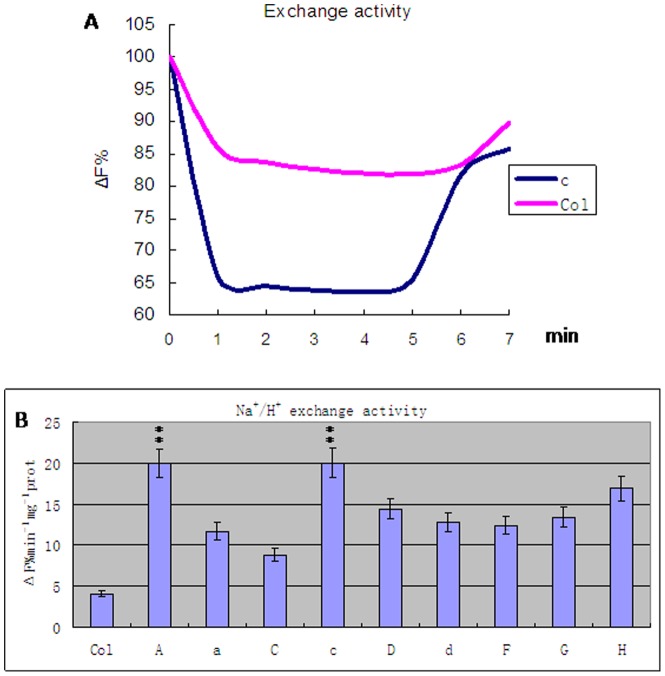
Na^+^/H^+^ antiport activity in transgenic carrying A, a, C, c, D, d, F, G and H subunit from wheat and wild type *Arabidopsis thaliana* plants (Col). A. The accumulation of protons inside the vesicles is determined through acridine orange Xuorescence quenching. B. The activity of Na^+^/H^+^ antiport activity. Values are means ± SD (n = 5 independent experiments) (ANOVA test, P<0.01).

Na^+^/H^+^ exchange activities were tested by Na^+^ absorption and H^+^ exsertion (Fig. 6, Fig. S3). The initiation of H^+^-ATPase activity by ATP would quench fluorescence. When this quenching reached a stable level, the addition of Na^+^ would restore fluorescence in a dose-dependent manner. The results indicate that transgenic plants had significantly higher Na^+^/H^+^ exchange activities than the controls (ANOVA test, P<0.01) (Table 1). Plants with the c or A transgene had activities fourfold that of the control plants (Least significant difference test, P<0.01) (Table 2). Plants with the C subunit transgene had the smallest increase in activity, which was about twofold greater than that of the control (Least significant difference test, P<0.01) (Table 3). Plants with another transgene had activity about threefold higher than that of the control plants (Least significant difference test, P<0.01) (Table 4).

**Table 1:** ANOVA test results of V-H^+^-ATPase Activity.

Source	Degrees of freedom	Sum of Squares	Mean Square	F Value	F_0.05_	F_0.01_
Gene	9	1636073.74	181785.97	40.48^**^	2.12	2.89
Error	40	179625.86	4490.65			
Total	49	1815699.60				

**Table 2:** Least-significant different tests of V-H^+^-ATPase Activity.

Gene	Average	significance level	
		α = 0.05	α = 0.01
c	1068.64	a	A
A	1017.03	a	A
H	729.6	b	B
G	716.09	bc	B
D	710	bc	B
a	663.98	bc	B
d	655.57	bc	BC
F	643.12	c	BC
C	548.16	d	C
Col	447.05	e	C

**Table 3:** ANOVA test results of Na^+^/H^+^ exchange Activity.

Source	Degrees of freedom	Sum of Squares	Mean Square	F Value	F_0.05_	F_0.01_
Gene	9	1063.22	118.14	69.93^**^	2.12	2.89
Error	40	67.58	1.69			
Total	49	1130.80				

**Table 4:** Least-significant different tests of Na^+^/H^+^ exchange Activity.

Gene	Average	significance level	
		α = 0.05	α = 0.01
c	19.98	a	A
A	19.88	a	A
H	16.86	b	B
D	14.39	c	BC
G	13.34	cd	C
d	12.75	cd	C
F	12.34	cd	C
a	11.65	d	C
C	8.73	e	D
Col	4.02	f	E

### Analysis of V-H^+^-ATPase Subunit Expression Mode

**Figure 7:**
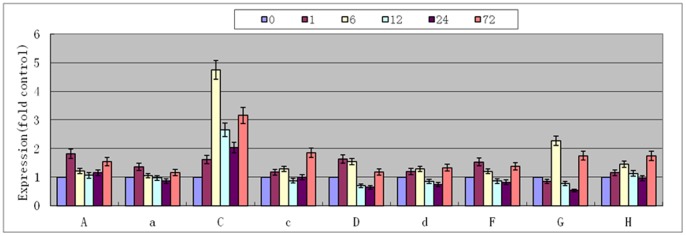
Relative expression of V-H^+^-ATPase subunits in *Arabidopsis thaliana* under 170 mM NaCl stress for 0, 1, 6, 12, 24 and 72 h. Values are means ± SD (n = 5 independent experiments) (Student’s t-test, P<0.05).

Quantitative analysis results showed that the expression of V-H^+^-ATPase subunits in *Arabidopsis thaliana* was increased after treatment with 170 mM NaCl. The C subunit increase was the greatest, and most sensitive to salt. After 6 h of treatment, the level of expression was 4.7-fold higher than that before the treatment (Fig. 7). Then, the expression decreased until 72 h, at which time the expression was 3.2-fold that before the experiment (Student’s t-test, P<0.01). The expressions of A, a, D and F subunits were highest after 1 h of treatment, being 1.8-, 1.3-, 1.6- and 1.5-fold that at 0 h. Thereafter, the expressions decreased. After 72 h, the expressions were 1.2- to 1.5-fold that before treatment (Student’s t-test, P<0.05). The expressions of d, G and H subunits were the highest after 6 and 72 h (Student’s t-test, P<0.05). The expression of the c subunit was the least sensitive to salt, and only increased by 1.8-fold after 72 h (Student’s t-test, P<0.05).

## Discussion

Compartmentalization of Na^+^ in vacuoles is one of the three strategies employed by plants to increase salt resistance [25]. Compartmentalization of Na^+^ mainly depends on the Na^+^/H^+^ exchange activity at the plant vacuolar membrane. This exchange is secondary active transport, which is powered by a proton gradient. V-H^+^-ATPases hydrolyze ATP and pump proton into vacuoles, which forms a cross-membrane proton gradient, which in turn provides energy for Na^+^ compartmentalization [26]. Therefore, V-H^+^-ATPases play an essential role in plant salt resistance. We cloned all 11 subunits of wheat V-H^+^-ATPase and subjected them to further study.

We first examined the expression of each subunit in wild-type *Arabidopsis thaliana* under salt stress. The results indicated that the expression of the c subunit was not significantly altered at the beginning of salt stress, and only increased after 72 h (Fig. 7). The c subunit was the least sensitive, with delayed elevation of expression under salt stress. These findings suggest that the c subunit is a factor that limits the full enzyme activity. In addition, the sequence of the c subunit was found to be most conserved among V-H^+^-ATPase (Fig. 2), which suggests that it is a limiting factor for enzyme function. The expression of the C subunit was rapidly increased under salt stress (Fig. 7), which suggests that this subunit is the most sensitive to salt stress and that it is not a limiting factor for enzyme activity. The remainder of the subunits showed various levels of elevated expression under salt stress, with peak levels occurring around 1 to 6 h, which suggests that these subunits have certain levels of sensitivity and play certain roles in enzyme functions. In order to verify these hypotheses, we made transgenic *Arabidopsis thaliana* for these subunits and tested the salt tolerance of these transgenic plants. The results indicated that plants with the c subunit transgene showed the greatest increase in salt tolerance, while those containing the C subunit transgene showed the least salt tolerance, with the other transgenic plants being intermediate increases in salt tolerance (Fig. 3, 4). These observations were consistent with thehypotheses.

The results of the V-H^+^-ATPase activity test further supported our hypotheses. Plants with the c subunit transgene showed the highest V-H^+^-ATPase activity, those with the C subunit the lowest (Table 1, 2), with the remainder somewhere in-between (Fig. 5). These observations may be directly related to the functions of these subunits. The functional enzyme is assembled from these subunits at various proportions and each of these subunits has a different role in the assembled enzyme. The c subunit hexamer forms the V0 core as part of the ion channel, participating in the cross-membrane ion transport [27]–[28]. Overexpression of the c subunit may allow more proton channels to form and increase the activity of V-H^+^-ATPase. The A subunit catalyzes the hydrolysis of ATP, providing energy for proton transportation. The D subunit forms the central axis of the V1 domain [29], which is necessary for ion transport and ATP hydrolysis [30]. The G subunit is a component of the V1 stalk, participating in the coupling of ATP hydrolysis and H^+^ transport. The C, F and H subunits participate in the stabilization of V1 and its connection with V0. The d and c subunits have close contact with each other and stabilize V0 assembly. The a subunit is the largest subunit in V-H^+^-ATPase and may play a role in its assembly and localization [26]. Overexpression of the A, a, D, d, F, G and H subunits contributes relatively little to the enzyme activity.

In order to understand the mechanism of salt tolerance, we examined Na^+^/H^+^ exchange activities in *Arabidopsis thaliana* plants carrying a transgene of one of the V-H^+^-ATPase subunits, and compared these activities with those in wild-type *Arabidopsis thaliana* plants (Fig. 6). The results showed that plants with the c subunit transgene had the greatest increase in Na^+^/H^+^ exchange activity, with those with the C subunit showing the least increase, and all other subunits in-between these two extremes (Table 3, 4). These results demonstrate that the higher the V-H^+^-ATPase activity, the higher the cross-membrane proton gradient, which in turn produces a greater proton driving force. In turn, more Na^+^ is compartmentalized in vacuoles, and hence there is higher salt tolerance.

In summary, under salt stress, plant V-H^+^-ATPase activities increase, which in turn increases the cross-membrane electrochemical gradient. As a result, salt is compartmentalized in vacuoles, which ensures normal development. This is probably a very important strategy for salt tolerance in plants. Since the c subunit is the limiting factor of V-H^+^-ATPase activity, its use as a transgene in plants may enable better salt tolerance to be achieved.

## Supporting Information

Figure S1 **Overexpression of wheat V-H^+^-ATPase c subunit in transgenic plants was confirmed by RT-PCR.**
(TIF)

Figure S2 **Activity of V-H^+^-ATPase in transgenic and wild type Arabidopsis thaliana plants (Col).**
(TIF)

Figure S3 **Na^+^/H^+^ antiport activity in transgenic and wild type Arabidopsis thaliana plants (Col).**
(TIF)

Table S1 **Primers used in PCR.**
(TIF)

Table S2 **Primers for vector construction.**
(TIF)

Table S3 **Primers for Real-time quantitative RT-PCR.**
(TIF)
