# Correction: Wheat V-H^+^-ATPase Subunit Genes Significantly Affect Salt Tolerance in *Arabidopsis thaliana*

**DOI:** 10.1371/journal.pone.0098728

**Published:** 2014-05-27

**Authors:** The PLOS ONE Staff

The affiliation for the first author is incorrect. Xiaoliang He is not affiliated with #2 but with #1 College of Life Science of Hebei Normal University, Shijiazhuang, Hebei, China. Xiaoliang He’s current address is as follows: School of Biological Science and Engineering, Hebei University of Science and Technology, Shijiazhang, Hebei, China.
