# Supplementary figures and images for: Wheat V-H+-ATPase Subunit Genes Significantly Affect Salt Tolerance in Arabidopsis thaliana
Source: PLoS One. 2014 Jan 30;9(1):e86982. doi: 10.1371/journal.pone.0086982 (PMC3907383; doi:10.1371/journal.pone.0086982)

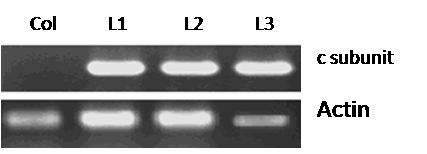

Supplement: Figure S1 — Overexpression of wheat V-H+-ATPase c subunit in transgenic plants was confirmed by RT-PCR. (TIF) [file pone.0086982.s001.tif]

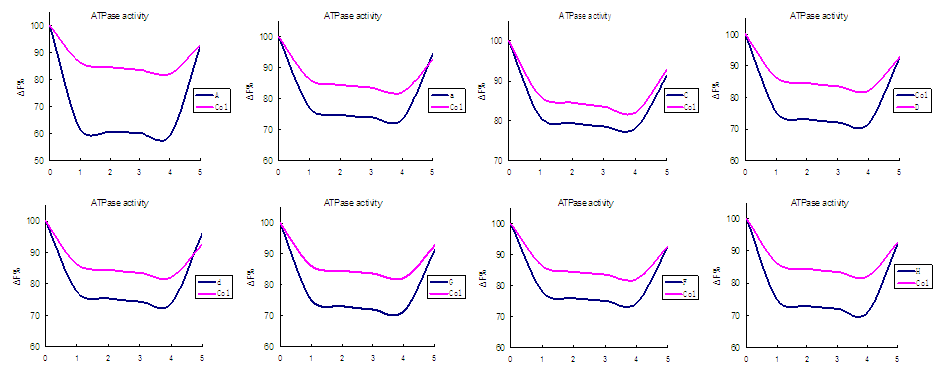

Supplement: Figure S2 — Activity of V-H+-ATPase in transgenic and wild type Arabidopsis thaliana plants (Col). (TIF) [file pone.0086982.s002.tif]

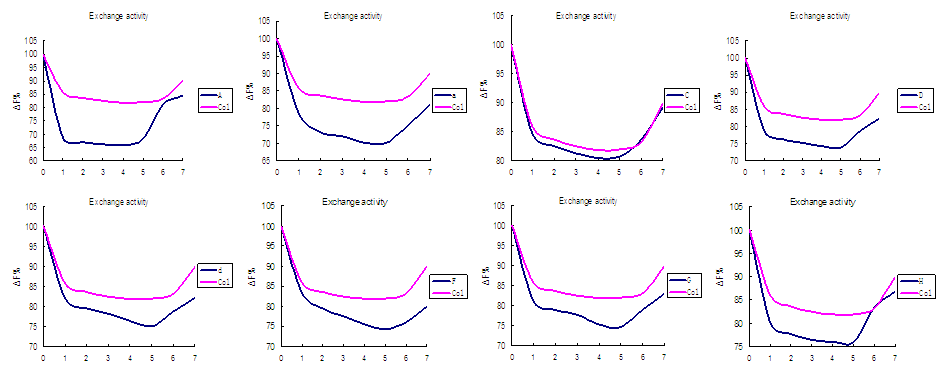

Supplement: Figure S3 — Na+/H+ antiport activity in transgenic and wild type Arabidopsis thaliana plants (Col). (TIF) [file pone.0086982.s003.tif]

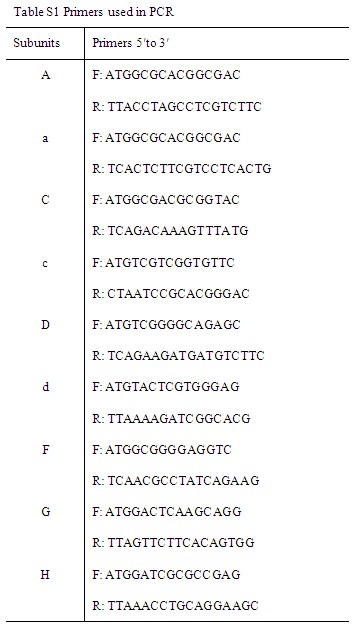

Supplement: Table S1 — Primers used in PCR. (TIF) [file pone.0086982.s004.tif]

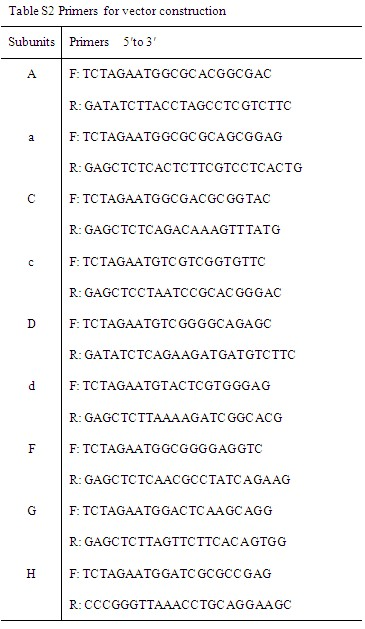

Supplement: Table S2 — Primers for vector construction. (TIF) [file pone.0086982.s005.tif]

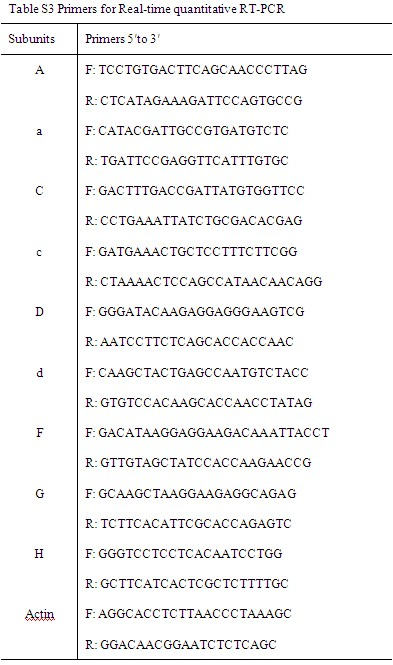

Supplement: Table S3 — Primers for Real-time quantitative RT-PCR. (TIF) [file pone.0086982.s006.tif]
